# Physiology

**Published:** 1860-05

**Authors:** S. Weir Mitchell

**Affiliations:** Lecturer on Physiology in the Phila. Med. Association


### PHYSIOLOGY.

BY S. WEIR MITCHELL, M.D.,

LECTURER ON PHYSIOLOGY IN THE PHILA. MED. ASSOCIATION.

### I.—*Remarks on a Critique of Dr. Brinton's on my Memoir on a little-known Function of the Pancreas.* By LUCIEN CORVISART, M.D., Paris.*

Dr. Brinton has done me the honor to submit to the test of his own experiments† my researches‡ on the pancreas, or rather some points in these researches. Dr. Brinton's work would have had more weight, if the author had not shrunk from repeating the vivisections from which I have derived so much information;

---

* Translated for M. Corvisart, from his manuscript, by William Daniel Moore, M.B., M.R.I.A.

† See the number of this journal for August, 1859.

‡ Sur une fonction peu connue du Pancréas, etc., par Lucien Corvisart. Paris, Victor Masson. 8vo., pp. 123.

and above all, if before undertaking or recording his researches, he had been more fully acquainted with the medical literature of the subject. In fact, at the period at which Dr. Brinton published his observations, Professor Meissner* had, under the title of "Investigations as to the Digestion of Albuminous Bodies,"† completely confirmed the fact of the energetic digestive action of the pancreas on albuminous aliments, which it would have been well to have repeated. Moreover, I had pointed out, in a short notice,‡ the gross error which had misled MM. Keferstein and Halwachs. It is well known that Montègre, in studying the gastric secretion, *always sought in vain* for the gastric juice, because he always sought it in the fasting state, and that by this obstinate course he was led to deny the existence of the gastric juice, which nevertheless thrust itself forward before the eyes of all. MM. Keferstein and Halwachs, my opponents, falling into an error of the same kind, had rendered themselves powerless, and had, so to speak, concealed from themselves the action of the pancreas, by forgetting that it is not during fasting and repose that the gland must be taken, to find it in its ferment and its activity, but during the very act of digestion, (five or six hours after a meal in the dog.) But if I thus dwell upon publications anterior to that of Dr. Brinton, it is because he, as well as Montègre and MM. Keferstein and Halwachs, has, without knowing it, very evidently divested most of his observations of any force. I had taken some pains to spare experimenters the annoyance of the troublesome groping through which I had to pass, and to enable them to avoid negative or doubtful facts, like those of Dr. Brinton. "It is necessary," I said,§ "in preparing an infusion of the pancreas for the purposes of study, to avoid *crushing* the gland, or agitating it too frequently in the water, or protracting the infusion beyond the period when the fluid becomes clouded." I had even taken care to point out this state as an indication for rejecting beforehand infusions, which might have attained to opalescence, as inert, and unfit for the search after positive results. "Commonly," I added, "an infusion which is long filtering and is cloudy, has partly lost its efficacy. * * * * The turbidity shows that the pancreatic juice is beginning to act upon the fatty matters in the gland itself; at a later period it will have begun to act upon the azotized substance, since, like the gastric juice, the pancreatic fluid exhausts itself by agitation.|| Taken in this state, the infusion of the pancreas would no longer exhibit to the experimenter any digestive action.¶ Now these very unfavorable

* Zeitschrift für Rat. Med., 1859, dritte Reihe, Band viii.

† Untersuchungen über die Verdanung der Euiveisskörper.

‡ The Lancet, June 18, 1859, and Union Médicale, 23 July, 1859.

§ Lancet, June 18, 1859, page 607.

|| Lancet, loc. cit.

¶ Facts are no doubt to be found in Canstatt for this year, due to Professors V. and S., which are completely confirmatory of another action described by me (Lancet, loc. cit.,) and which will much surprise physiologists: when the pancreas is taken from a dog five hours after a meal, the juice contained in it is so energetic, that if the infusion of the gland be a little too prolonged, if the latter have been cut up in very small pieces, and, above all, if it be too often and violently agitated, the temperature being maintained at 104° F., the gland in part disappears, dissolved and digested in its own juice, which has escaped from the channels where normally it is confined during life.

conditions, which I have pointed out in order that they might be avoided, Dr. Brinton has selected and observed; and, what is more, he congratulates himself on having done so!

"The method I generally adopt is to mince a given weight of pancreas very finely, before pounding it to a pulp in a porcelain mortar. Twice or thrice as much distilled water is then gradually added to and rubbed down with the pulpy mass; the whole is then filtered, so as to yield an opalescent (almost milky-looking) fluid."[?] "This process is in many respects superior to that of Corvisart (Brinton.) Thus we see the application of the adage, 'le mieux est quelquefois l'ennemi du bien.'" The process followed by Dr. Brinton, and which, before him I had pointed out, not as a process, but as a mischance, is therefore fit only to show that it is possible in an instant, either in an entire pancreas or in half a pancreas, to exhaust all digestive power on the albuminoids by *pounding it;* which complete or partial exhaustion is recognized even before the digestive experiment, by the fact that the liquid is milky and filters persistently turbid—facts which I had expressly stated, as has been seen. The precaution I adopted of taking the pancreas immediately after the expiration of the fifth hour after a meal, of avoiding, before and after death, bruising or pounding the gland or protracting the infusion beyond a period easily determined by observation, had led me to the discovery of facts invariably as similar as physiological facts usually are. It will be observed that Dr. Brinton's unfortunate deviation from this mode has led him on the contrary to fluctuate incessantly between my views and those of my opponents. At one time Dr. Brinton writes: "sometimes we may see no solution at all;" at another he declares that a "rapid, energetic solution occasionally occurs." In one place he writes: "the solution is accompanied with unmistakable putrefaction;" in another, on the contrary, he says, "that an energetic solution is often seen to occur when as yet none but a peculiar, penetrating, quasi-syrupy odor is discernible." Now he declares, with me, that the solution is "rapid;" again he considers it to be "slow." Such are the uncertainties Dr. Brinton would have been spared, if he had been acquainted with the publications which have preceded his experiments. Dr. Brinton seems, moreover, in the second place, to have made the same mistake as MM. Keferstein and Halwachs, in not choosing for his experiments the pancreas taken the fifth hour after a meal. Lastly, I may in the third place add, that although Dr. Brinton appears to trace a certain connection between putrefaction and digestion, all the observations he has made, and in which some putrefactive odor, however slight, was developed, will never be considered by me—nor, I think, by any physiologist—as cases of digestion. I would particularly reject the experiment where, having proved the digestive inefficacy of the pancreas, he allows the fluid to putrefy during five whole days, then places it in contact with albumen, and pronounces that there was subsequent solution, and that the digestive power was increased by the putrefaction. The experimenter should always stop before there is any odor or possibility of putrefaction.* In this latter fact I see only two things:—

* The pancreas is one of the organs most liable to putrefaction, and this is an additional reason for vigilance in making experiments on the digestive action of its

1. In the first instance, the pancreas, taken doubtless in the fasting state, (nothing is specified on this capital point,) is inert.

2. It was allowed to putrefy, and then communicated its putrescent liquefaction to the surrounding matters.

To recapitulate:—

1. Let Dr. Brinton never protract his experiments on artificial digestion beyond the period when the odor is the odor sui generis of the pancreas taken at the very moment of killing the animal.

2. When he meets with an inert pancreas, let him not seek in putrefaction an aid to solution.

3. Let him never crush the pancreas.

4. Let him reject beforehand as inert any infusion which passes through the filter turbid and lactescent.

5. Let him never take a pancreas except at the time I have thought it right to indicate.

6. Let him prefer the pancreas of a carnivorous animal, like the dog, when the periods of digestion are well marked.

7. Let him repeat the vivisections, which he has very gratuitously refused to do, and without referring to the confirmatory experiments of Professor Meissner, or those witnessed by MM. Kühn, Snellen, Professors Moris, Schiff, etc. I have more than a hope that, disengaged from a false starting-point, the experiments instituted by Dr. Brinton's able and methodical intellect will quickly and decidedly convince him as to the point in my memoir which has appeared to him interesting. Moreover, I earnestly invite experimental criticism of others; whether they be regarded with favor or disfavor, they are closely connected with the physiology of digestion, and from the courteous collision of opinions, however evoked, the truth will be elicited.—(*The Dublin Quarterly Journal of Medical Sciences*, February, 1860.)

II.—*Notes on some Experiments on the Cervical part of the Sympathetic Nerve of a Beheaded Woman.* By Professor RUDOLPH WAGNER.

"This morning at half-past nine o'clock a woman twenty-eight years old was beheaded, at a short distance from our town, and I made some experiments upon her head, which confirm and complete a number of experiments of the same character made last summer, and referred to in the first series of my researches upon the functions of the brain. The principal object of these researches was to determine the function of the smooth muscular fibres recently discovered by Dr. Müller of Wurzburgh."

---

juice or of its infusion. At the temperature of 104° F., the digestion of fibrin is usually accomplished without putrefaction in the third hour; that of albumen in the eighth, if the pancreas has been taken out and the infusion made within the preceding four hours. One hour's infusion at 104° F. suffices to remove from the gland all its juice. If the pancreas and its infusion be older, or if the digestion be more protracted, the experimenter is at fault, and allows the almost fatal supervention of putrefaction.

Any infusion of the pancreas which requires more than an hour's digestion at 104° F., ought to be considered as inert.

After describing certain circumstances which interfered somewhat with the examination, Professor Wagner goes on to say:—

"The decapitation took place at thirty-two minutes after nine o'clock, and eighteen minutes afterwards, namely, at fifty minutes after nine o'clock, the head was put upon the dissecting table. The head had been carried to the laboratory wrapped in a warm cloth.* The knife had cut the sixth cervical vertebra, and the division of the two great sympathetic nerves had occurred about an inch and a quarter below the swelling of the superior cervical ganglion. The temperature of the interior of the buccal cavity was at 99° 32 Fahr. The inferior portions of the neck were still warm. The palpebral opening was about five millimetres; the pupils were of middling size, (about five millimetres in diameter.) The axes of the eyes were horizontal and parallel to one another.

To produce the excitation I had prepared two electro-magnetic rotatory apparatuses, on account of the ease with which they can be carried and used. The sympathetic nerves when prepared were put in contact with the platinum needles of the reophore, in accordance with the rules of Dubois-Reymond.

The eyelids having been closed by gentle pressure, electrical stimulus was applied to the cervical portion of the right sympathetic. The eyelids opened slowly about three or four seconds after the apparatus was put in action. There was especially a marked lifting of the upper eyelid. The vertical diameter of the palpebral opening reached to about eight millimetres, but later decreased to six millimetres. Between the eyelids the lower half of the cornea was visible, behind which the pupils could be seen to dilate. When the electrical stimulus was very strong, the dilatation of the pupils became so great that the iris was hardly from one and a half to two millimetres in size.

The same experiment produced like results upon the other eye, and indeed the experiment was repeated six times upon one side until twenty-five minutes had elapsed since the beginning of the experiment, and consequently three-quarters of an hour after decapitation. It was even possible to attain to like success when the ends of the sympathetic, separated from the surrounding tissues, were almost cold. The eyelids then opened more slowly; about six seconds elapsed between the beginning of the action of the apparatus and the movement of the eyelids, which, in order to insure success, we were obliged to close entirely before attempting the experiment.

After it became impossible to succeed any longer by applying the conductors near the cut end of the nerve, a small opening was obtained, after about thirty minutes, by applying the conductors to the ganglion itself, then exposed for the first time. The iris continued irritable some time longer. The dilators of the pupil were still irritable at the seventh experiment on the same side and obeyed the stimulus of the great sympathetic at the thirtieth minute. They would have responded still later had not the head been taken away by Henle for the purpose of experiment.

---

* It is remarkable that the eminent physiologist, to whom we owe this labor, should still remain in ignorance of the law, that the lower the temperature of the nervous and muscular tissues, the longer they retain their vital properties. According to this law, Wagner could have done nothing more injurious than to prevent the cooling of the head upon which he was about to experiment.

No change in the curvature of the cornea was noticed, nor even any of that lifting of the ocular globe so plainly observable in experiments on animals. (See *Neurologische Untersuchungen*, p. 132.) In the dog I could estimate this rising up of the globe as equal to at least from two to four millimetres.

There remains no doubt that in these experiments the opening of the eyelids does not depend on the elevator of the superior eyelid as has been recently asserted, since the movement has really all the characters which belong to the action of smooth muscular fibres in accordance with the laws enunciated by Edmund Weber; thus a perceptible interval elapses between the beginning of the excitation and the contraction, its visible effect; and this contraction, moreover, persists, after this excitation has ceased. When the extremities of the reophores were carried deeply into the muscles of the neck, and excited at one and the same time, branches of the hypo-glossal nerves, the facial and the fourth pair, there were produced tremulous movements in the muscles of the chin, the mouth, the nostrils, and of the jaws, as well as in the orbicularis muscle of the eyelids.

The well-known twitching of the muscles thus produced by galvanism differed totally from the slow movement of the eyelid caused through the agency of the great sympathetic.—(*Journal de Phys.*, January, 1860; from *Zeitschrift für Rationelle Medizin*, dritte R. Band v., 1859.)

III.—*On the Existence of Certain Smooth Muscular Fibres in the Orbit of Man and Mammals.* By H. MULLER.

1. The inferior orbital fissure in man contains a mass of reddish-gray color, composed of hundreds of smooth muscular fibres, the most of which are provided with elastic tendons.

2. In mammals a more strongly developed muscle is found; as the analogue which we have just described, it forms a skin muscle—the orbital muscle of some writers, and is equally composed of smooth muscular fibres.

3. The nictitating muscle is partly composed of smooth muscle and partly of tractile fibres transversely striated, acting from in front backward.

4. The orbital muscle is provided with bundles of nerves which contain scarcely any fine fibres, such as characterize the sympathetic system. These nerves are partly derived from the spheno-palatine ganglion.

5. The orbital muscle is the agent in producing that forward movement of the ball of the eye, observed in animals during excitation of the cervical sympathetic; it serves to antagonize those muscles which draw or push the eye toward the bottom of the orbit.—(*Journal de la Physiologie*, January, 1860; *Trad. de Zeitschrift für Wissenschaftliche Zoologie*, Band x., Haft 4, 1858.)

IV.—*Existence of Urea in the Fluids of the Thoracic Duct.* By M. WURTZ.

M. Wurtz has discovered that urea is one of the principal constituents of the fluids of the thoracic duct. Guided by the idea that urea is probably formed not in the capillaries but in the extra-vascular tissues themselves, the author sought for this substance in the chyle and the lymph.

In a dog nourished with meat, one thousand grammes of blood yielded 0·089 of urea; and the same quantity of lymph, 0·158 of urea.

In a cow the same amounts of blood, of chyle, and of lymph, gave respectively 0·192, 0·192, 0·193, as the amount of urea present.

The blood and chyle of a ram, contained in each one thousand grammes, respectively, 0·248 and 0·280 of urea.—(*Comptes Rendus de l'Acad.*, July 4, p. 52.)

